# Does FDI foster technological innovations? Empirical evidence from BRICS economies

**DOI:** 10.1371/journal.pone.0282498

**Published:** 2023-03-09

**Authors:** Najabat Ali, Khamphe Phoungthong, Anwar Khan, Shah Abbas, Azer Dilanchiev, Shahbaz Tariq, Muhammad Nauman Sadiq

**Affiliations:** 1 Environmental Assessment and Technology for Hazardous Waste Management Research Center, Faculty of Environmental Management, Prince of Songkla University, Songkhla, Thailand; 2 Faculty of Management Sciences, Hamdard University, Islamabad, Pakistan; 3 School of Economics, Xiamen University, Xiamen, China; 4 Gongqing Institute of Science and Technology, Gongqing, China; 5 Faculty of Business and Technologies, International Black Sea University, Tbilisi, Georgia; University of Sargodha, PAKISTAN

## Abstract

The idea behind the spillover effect of FDI on economic growth is based on the idea that multinational companies can bring technological innovation and rich knowledge to host countries. Therefore, FDI plays a vital role in technological innovations. This study aims to investigate the impact of foreign direct investment (FDI) on the technological innovation of BRICS countries from 2000 to 2020. This study uses the latest econometric techniques, such as the cross-sectional dependence (CD) test, second-generation unit root tests, panel cointegration tests and the Dumitrescu-Hurlin causality test. For long-run run estimation, this study uses the augmented mean group (AMG) panel estimator and the common correlated effects mean group (CCEMG) estimator for empirical analysis. The findings of the study show that foreign direct investment (FDI), trade openness, economic growth, and research & development expenditure positively impact technological innovation in BRICS countries. Also, the model’s long-term causality and lagged error correction term (ECT) are significantly negative. Suggested policy measures will be helpful for BRICS economies in boosting technology innovation through FDI.

## 1. Introduction

Foreign direct investment has been essential for business expansions and cross-border knowledge flows, mainly in developing countries during the last three decades. FDI from developing countries substantially impacts technological innovation, improving organizational and managerial skills and stimulating domestic investment [1]. On the other hand, due to globalization, multinational companies face intense competition to increase their global trade share through market competitiveness. The competitiveness of today’s world is producing diversified and innovative products (i.e., high-tech products). In the modern world, the determinants of competitiveness are not factor endowments or accumulation of natural resources but research and development (R&D) and technological innovations [2, 3]. The dynamic nature of global competitiveness convinces firms to effectively enhance their technologies and products to compete in the international market. Under such circumstances, technological innovation’s role has become increasingly important for competitive economic growth and development in the current era. Therefore, the government will inevitably benefit from domestic companies producing innovative products or foreign direct investment (FDI) flows into multinational companies with high-tech production facilities. More than 90% of production technology comes from foreign sources in low- and middle-income economies [4, 5]. Among other channels, FDI is often used to transfer new technologies across borders. In the past two decades, FDI has gained widespread recognition in high-tech products by supporting the host country’s innovation and development (R&D) expenditure [6, 7].

Through the transfer of financial capital, technological innovation, and management expertise, FDI plays a considerable role in economic growth and development [8]. It will also stimulate technology spillovers, promote human resource development, help realize international trade integration, create a competitive environment, and contribute to enterprise development [9]. In the past few years, FDI has excelled in foreign trade because the production flow is higher than foreign trade goods’ inflow. With the increase in global international trade protection, FDI has become a massive opportunity for many companies to enter other countries’ markets directly. Therefore, FDI has become prominent due to its technology spillover effects [10–12]. The importance of FDI in bringing technological innovation into the world, especially in developing economies, is increasing, encouraging multinational companies to invest in host countries [13–15]. The inflow of FDI brings financial capital, new technology and knowledge. Also, FDI promotes the manufacture of high-tech and high-quality products. It has also contributed to the production of value-added export products through multinational companies’ participation. The R&D expenditure of multinational corporations is usually high, promoting technological innovation and bringing modern technology into the host country. Consequently, the transfer of production processes and technologies from developed countries to developing economies is achieved through FDI [16].

Various studies have used patent applications as a proxy for technological innovation [2, 17–20]. Therefore, researchers expect that a rise in FDI will increase the number of patent applications. It is generally believed that FDI has brought modern technology and new products, stimulating technological innovation in recipient countries [21, 22]. Various experts such as engineers, scientists and technicians help in technological advancement. Technological innovations promote advanced knowledge from research and development experiences [23]. It is an assumption that research and development expenditure positively affects technological innovations in host economies. There are sufficient funds, better infrastructure, and a feasible investment environment in research and development in developed nations, so their innovation capability is higher than others. A country’s economic growth and development and its level of welfare are expressed in per capita GDP. Therefore, GDP per capita growth is also assumed to lead to the host country’s economic innovation growth [20, 24].

Recently Sugiharti et al. [25] investigated the effects of FDI spillovers on output and productivity in Indonesia’s manufacturing industry from 2010 to 2015. Results revealed that FDI benefits all technological groups through productivity and efficiency improvement, with high-tech firms seeing the most benefit because they have a strong ability to adopt the technology. Despite showing lower technical efficiency levels, tech firms in other categories also gain advantages from FDI through their ability to adopt new technologies. Similarly, Vujanović et al. [26] analysed the influence of FDI spillovers on the different stages of the innovation process, specifically for firms that utilize and generate knowledge in a developing European economy. The results suggest that in developing economies, innovation is more likely to involve copying rather than developing new products. Companies in the local area gain advantages from FDI in the initial stages of the innovation process, and the effects are more pronounced among firms that innovate by utilizing knowledge rather than creating knowledge. Liu et al. [27] also investigated the impact of FDI on regional innovation and the underlying mechanisms in China by using a “spatial autoregressive model” and a “threshold regression model” on a panel dataset of 253 cities spanning from 2003 to 2017. The results indicated that FDI significantly influences regional innovation and generates positive spillovers among cities. Additionally, the study found that innovation is typically concentrated in certain regions, and the flow of technology and knowledge from FDI facilitates the coordination of innovation and development among neighbouring cities. Another study by Yue [28] shows that FDI can boost the innovation capabilities of local Chinese businesses by way of spillovers and competition effects; however, the effects may differ among local firms. The most significant impact is observed in non-state-owned enterprises with high productivity and capital intensity. Additionally, Song and Han [29] studied the influence of FDI on green innovation efficiency. This study found that while FDI has a more negligible inhibiting effect on green innovation efficiency than a promoting effect, the overall effect is not positive due to regional differences. Additionally, the study suggests that due to China’s current imperfect economic structure, regions have not yet been able to utilize FDI for green innovation fully. To improve this, the study suggests developing green innovation markets, incentivizing foreign companies with green technology to strengthening the connection between FDI and green technology innovation in China. While examining the drivers of inclusive growth in top African nations, Wang et al. [30] finds that technology adoption, trade openness and foreign investment positively impact inclusive growth for both individuals and society as a whole. The study recommends policymakers implement measures to advance technology, open markets to trade and attract foreign investment improve financial inclusion to support inclusive growth by creating more opportunities.

The BRICS countries have become fast-rising economies and have a substantial impact on the regional and global economies. The BRICS countries account for 41% of the world’s total population and more than a quarter of the region. Due to their important position in the world economy, the BRICS economies play a significant role in the economic growth of the world economy. Between 2000 and 2018, the GDP of BRICS increased from 8.5% to 23.58%, while exports and imports increased from 7.9% to 20.31% and 4% to 15.84%, respectively. The BRICS countries can attract and utilize large amounts of FDI. Between 2000 and 2018, the BRICS’ share of global FDI increased from 6% to 19% [31]. However, the FDI role depends on the absorptive capacity of the host country’s economy [32]. The BRICS countries have demonstrated significant technological progress and innovation in the past two decades. The growing share of high-tech exports supports technological development in the BRICS [33]. The above discussion emphasized the importance of FDI to the technological innovation of the BRICS. Therefore, this study explores FDI’s impact on technological innovation in the BRICS countries, including Brazil, Russia, India, China, and South Africa, from 2000 to 2020. The main objectives of this study are as follows:

- To empirically investigate the impact of FDI on the technological innovation of BRICS economies.
- To analyze the impact of trade openness, research and development and economic growth on technological innovations of BRICS economies.
- To provide policy recommendations on the technological innovation of BRICS economies based on the study findings. The rest of this article is organized as follows:

Section 2 provides a brief literature review, Section 3 presents data and methods, Section 4 provides empirical results and discussions, and the last section discusses the conclusion and policy recommendations.

## 2. Literature review

Various studies have used heterogeneous econometric models in the past few years to empirically test the links between FDI, trade openness, economic growth, R&D expenditure, and technological innovations. Looking at the available studies, this study thoroughly reviewed the relevant literature to ensure an in-depth understanding of the chosen variables and their interrelationship.

The idea behind the spillover effect of FDI on economic growth is based on the idea that multinational companies can quickly obtain technological innovation and rich knowledge [34–36]. Similarly, FDI leads to knowledge spillovers and helps domestic companies improve their technology through internal effects. This is shown that the existing literature has much empirical evidence to support FDI on technological innovation through spillover effects. The early understanding of how foreign capital affects host countries can be traced back to Hymer’s [37] “theory of foreign direct investment” (FDI). According to Hymer, FDI is not just a simple exchange of assets across borders but also includes international production. He argued that FDI represents the transfer of a bundle of resources, including capital, management, and technology, and can be understood as an international application of industrial organization theory. In addition to Hymer’s theory, various studies in the literature support the idea that FDI acts as a conduit for technology transfer. For example, Findlay [38] argued that foreign investment could stimulate technology improvement by providing domestic firms with the opportunity to perceive advanced technology. Wang [39] extended this work and further suggested that an increase in FDI can lead to more investments in human capital, thereby enhancing the potential for the host country to catch up with more advanced economies. The previous literature on the spillover effects of FDI can be traced back to the 1960s when MacDougall [34] studied the externalities of FDI and concluded that FDI created general welfare. After that, FDI has become a significant factor in technology transfer from developed countries to developing countries. However, the literature on the theoretical and empirical link between FDI and technological innovations is scant. Available literature has mixed conclusions, which may be unable to build any consensus between these variables; see Alfaro and Chen [40], Ali and Xialing [41], Antràs and Yeaple [42], Anwar and Nguyen [43], Ali and Hussain [44], Castellani and Zanfei [45], Harrison and Rodriguez-Clare [46], Ali and Shaheen [47], Kose et al. [48], Ali et al. [49], Wen et al. [50], Dinh et al. [51], and Gul and Imran [52].

The study of Borensztein et al. [32] analyzed the relationship between FDI and economic growth using data from 69 emerging economies from 1970 to 89; employing regression analysis, the study concluded that FDI plays a massive role in promoting technological innovation in the host countries. Similarly, Sultana and Turkina [3] examined the causal link between FDI and technology innovation over 2009–2016 in a regression framework and obtained a positive correlation between FDI and technological innovations. Further, the study of Gorodnichenko, Svejnar, and Terrell [53] explored the links between FDI inflows, trade, and innovation on the firms and industries of 18 countries, and they obtained a significant positive impact of the FDI inflows in the local firms in emerging markets. Furthermore, they found that technology spillovers from FDI and trade are limited to firms directly related to foreign investment. In a similar study, Erdal and Göçer [2] tested the FDI innovation linkages of some emerging economies in Asia from 1996 to 2013. The study found a positive association between FDI and technological innovation in the recipient country’s economy through panel cointegration and causality techniques. Their study recommended that countries facing funding shortages and technological gaps should attract more FDI to fill this gap. Narula and Pineli [54] studied the spillover effect of multinational corporations and concluded that multinational corporations benefit host economies’ technological development. Girma and Gorg [55] examined the spillover impacts of multinational corporations on the regional clusters within China’s regions. The study’s findings show a direct positive effect of multinational corporations on local firms in China; however, the indirect impact was negative.

Using a general equilibrium model of three economies, Lin and Lin [56] studied the welfare impact of technological innovations arising from FDI. The study found a positive effect of FDI on technological innovations. Likewise, Xu [57] employed US multinational corporations’ data, and the study recommends strong complementarity between FDI and human resources. The findings further reveal that the productivity rate of foreign inflows is far greater than local investments, and foreign investment is also a vital tool for technological transfers. However, technological transfer merely takes place effectively when there is a minimum stock of human resource threshold in the recipient economy. Alfaro and Charlton [58] analyzed the industrial sector data of OECD economies. The study results found substantial growth in the countries that depend on foreign sources for investment purposes. Moreover, the study’s findings were similar to the mainstream literature on the gains of FDI. Using the Poisson model, Khachoo and Sharma [59] investigated the FDI-innovations nexus of India’s manufacturing sector between 2000 and 2013. They concluded that FDI moderately impacts the innovation level in similar sectors. Though, the effect of the firm’s innovative performance in supply industries seems to be more robust. Additionally, Gorodnichenko, Svejnar, and Terrell [53] employed industry-level and firm-level data of large firms from eighteen economies and found that FDI and trade positively affect domestic firms through the spillover effect. Moreover, the findings show that FDI’s spillover effect on developed countries’ firms is more effective. Similarly, Cheung and Lin [60] employed provincial data on domestic patents in China for 1995–2000. The Ordinary Least Square method results show a positive association between FDI and technological innovation. These findings seem robust under cross-sectional data estimations and pooled data series for various innovations. Similarly, Ascani and Gagliardi [61] employed the knowledge production function approach (KPF) to explore the relationship between FDI and innovations of Italian manufacturing firms from 2001–2006. The results of this research illustrate a significant role of FDI inflows in stimulating technological innovations. The results of the study were found to be robust for observing the impact of FDI.

The study by Blomström et al. [62] argued that the additional contribution of FDI inflow depends solely on domestic firms’ adaptability. In short, the potential benefits of FDI can only be realized if domestic firms can acquire foreign technology and skills. Therefore, it is necessary to support and develop domestic firms before subsidizing international firms. Hsu and Tiao [63] analyzed the relationship between FDI and patents in Asia from 1985–2010. Results obtained using the OLS and System GMM methods show a positive relationship between FDI and patents. Sandu and Ciocanel [64] assessed European Union (EU) member states’ innovation using the EUROSTAT data from 2007–2012. The results show a positive relationship between R&D spending and technological innovation. Bhattacharya and Bloch [65] used regression analysis to examine the determinants of innovation and found that R&D expenditure and trade openness positively and significantly impact technological innovation. Furthermore, Avila-Lopez et al. [24] tested the Granger causality between per capita growth and technological innovation in 12 Latin American countries from 1996 to 2015. The results show a one-way causal relationship between per capita growth and technological innovation. It is also found that there is a two-way causal relationship between GDP per capita and technological innovation. The study concluded that per capita GDP promotes technological innovation. Likewise, Akcali and Sismanoglu [66] studied the correlation between GDP and R&D expenditure in 19 developing and advanced economies selected from 1990 to 2013. The results of panel data analysis show that the increase in R&D expenditure promotes technological innovations.

Based on the thorough discussion of the available literature, most past studies have not found reliable conclusions. Their results remain mixed or cannot provide satisfactory results due to the traditional set of econometric applications. The inconclusive empirical investigations may arise due to cross-sectional dependence, panel heterogeneity, or omitted variable effects. Therefore, it provides a vacuum to investigate the FDI-innovations relationship. To the best of our knowledge, no recent empirical study has examined the impact of FDI on BRICS economies’ technological innovations. Therefore, this study proposes to explore the FDI-innovations nexus of BRICS economies for 2000–2020.

## 3. Data, methodology and model specification

The data on chosen variables, including patent application proxied for technological innovations in line with previous studies [67], Foreign direct investments net inflows supported from [68]. While other variables, trade openness, Research and Development expenditures, and real GDP per capita, have been used as the explanatory variables in the study. The data on all the variables are drawn from the World Bank, except trade openness, which is downloaded from “United Nations Conference on Trade and Development (UNTCAD)” database. For more clarity, the detailed description and definitions of variables are presented in Table 1.

**Table 1:** Variables, measurement units and definitions.

Variables	Unit of Measurement	Definition	Source
Patent applications	Numbers	Patent applications (residents and non-residents)	World Bank
Foreign direct investment	Percent	Foreign direct investment, net inflows (% of GDP)	World Bank
Trade openness	Constant US dollars	“Total trade of goods and services measured in millions of constant US dollars”	UNTCAD
R&D expenditure	Percent	Research and development expenditure (% of GDP)	World Bank
GDP per capita	Constant 2010 US dollars	“Gross domestic product divided by midyear population”	World Bank

Hereafter, the study presents estimation techniques (methodology) and the data using the chosen dimension of variables. The basic model of our research is as follows.

$${PAT}_{it}=f({FDI}_{it},{TO}_{it},{R\&D}_{it},{GDPPC}_{it})$$ (1)

Where PAT represents the patent applications which are used as a proxy for technological innovations; FDI shows foreign direct investment; TO indicates trade openness; R&D denotes research and development expenditure; GDPPC represents economic growth; i shows countries, and t means time. For convenience, the study transforms the variables into a log form. Hence, we can re-write the above equation as follows:

$$ln{PAT}_{it}=\alpha_{0}+\beta_{1}{FDI}_{it}+{\beta_{2}TO}_{it}+\beta_{3}{R\&D}_{it}+{\beta_{4}GDPPC}_{it}+\mu_{it}$$ (2)

Where *α*_0_ and *μ*_*it*_ represent the constant term and the error term, respectively; *β*_1_, *β*_2_, *β*_3_ and *β*_4_ stand for undermined coefficients. Further, to the empirical analysis of the Eq 2, we have built robust estimation strategy, highlighted in the forthcoming sections.

### 3.1 Estimation techniques

**Fig 1:**
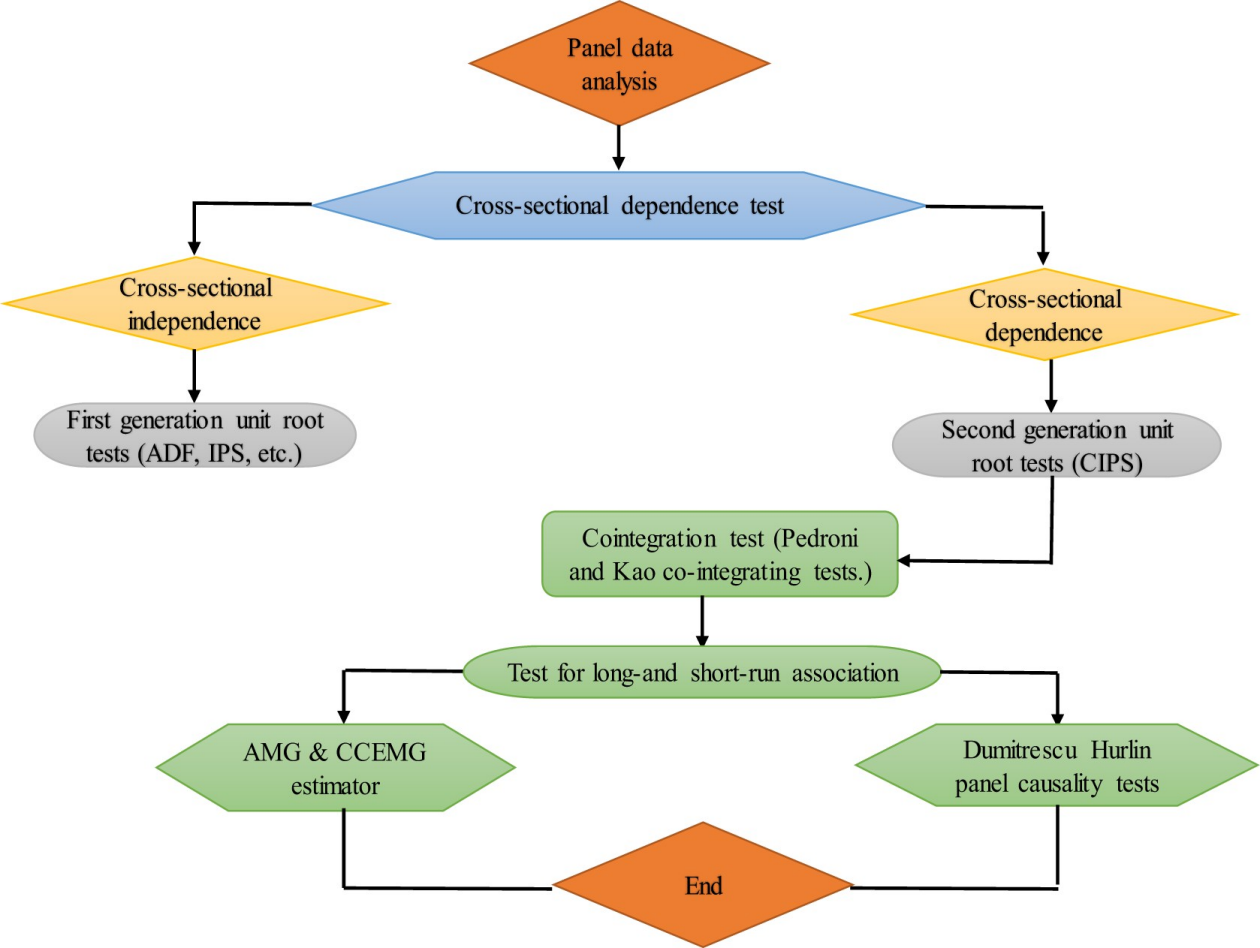
The estimation scheme for exploring FDI’s impact on BRICS economies’ technological innovations.

We present a systematic procedure for the empirical estimation of Eq (2). (i) We employed the Pesaran CD test for examining the cross-sectional dependence among the underlying variables in our model. (ii) For the unit root test, the study utilized the Pesaran CIPS test. (iii) Pedroni-cointegration and Kao-cointegration tests have been employed to observe integration among the variables. (iv) The study used the Augmented Mean Group (AMG) and CCEMG estimators to analyse the determinants of the patent application. CCEMG and AMG estimator is highly robust regardless of cross-sectional dependence and slope heterogeneity. The estimation scheme has been presented in Fig 1.

### 3.2 Cross-sectional dependence

To avoid any misleading results, there should be a cross-sectional correlation in the model [69, 70]. Therefore, in this study, we have used the Breusch-Pagan Lagrange multiplier (LM) test to check for cross-sectional dependence in our panel, and it is written as follows:

$${LM}_{1}=\sum_{i=1}^{N-1}\sum_{j=i+1}^{N}T_{ij}\partial_{ij}⟶\chi 2\frac{N(N-1)}{2}$$ (3)

However, for consistency, we have used Bias adjusted LMtest as well and presented it as under:

$${LM}_{2}=\sqrt{\frac{2T}{N(N-1)}}\sum_{i=1}^{N-1}\sum_{j=i+1}^{N}\frac{(T-k){{\hat{\partial }}^{2}}_{ij}-E(T-k){{\hat{\partial }}^{2}}_{ij}}{Var((T-k){{\hat{\partial }}^{2}}_{ij}}$$ (4)

Where ${{\hat{\partial }}^{2}}_{ij}$ indicates coefficients of correlation which were obtained from the above model. Eq (4) is distributed asymptotically as standard normal when the null hypothesis is considered *N*⟶ ∞ and *T*_*ij*_⟶ ∞.

### 3.3 Panel unit root test

After confirming cross-sectional dependence in the panel, the unit root tests of the first generation, such as Im, Pesaran, and Shin (IPS), Augmented Dickey-Fuller (ADF), Phillips- Perron (PP), seem to be invalid. Hence, we choose second-generation unit root tests, i.e., Pesaran Augmented Dickey-Fuller (CADF), and the Pesaran cross-sectionally Augmented Im Pesaran and Shin (IPS) tests. Pesaran [71] developed these tests. We can present the equation of CADF as follows:

$$\Delta X_{it}=a_{i}+b_{i}y_{i,t-1}+c_{i}{\bar{y}}_{t-1}+\sum_{j=0}^{k}d_{ik}\Delta {\bar{y}}_{t-j}+\sum_{j=1}^{k}d_{ik}\Delta y_{i,t-j}+\varepsilon_{it}$$ (5)

Where *y*_*i*,*t*−1_ and Δ*y*_*i*,*t*−*j*_ shows the first differences of each unit and the mean of lagged-level cross-sectional values. After the calculation of CADF, we can estimate the CIPS statistics as follows:

$$CIPS=N^{-1}\sum_{i=1}^{N}t_{i}(N,T)$$ (6)

Where *t*_*i*_(*N*, *T*) shows t-statistics in the CADF test define in Eq 5.

### 3.4 Panel co-integration test

We have used the Pedroni cointegration test for detecting cointegration in our panel. Pedroni [72] developed this test. The Kao [73] panel cointegration test has also been employed to avoid biased results. The panel-specific autoregressive (AR) test statistic and the same-specific autoregressive (AR) test statistic have been used for cross-sectional dependencies of the panel cointegration test.

We can express panel-specific-AR test statistic as follows:

$$VR=\sum_{i=1}^{N}\sum_{t=1}^{T}{{\hat{E}}^{2}}_{ij}{{\hat{R}}^{-1}}_{i}$$ (7)

We can estimate the same-AR test statistic as given below:

$$VR=\sum_{i=1}^{N}\sum_{t=1}^{T}{{\hat{E}}^{2}}_{ij}{\left(\sum_{i=1}^{N}{\hat{R}}_{i}\right)}^{-1}$$ (8)

Where *VR* indicates, the group mean-variance ratio statistics. ${{\hat{E}}^{2}}_{ij}=\sum_{t=1}^{T}{\hat{e}}_{ij},\;{\hat{R}}_{i}=\sum_{t=1}^{T}{{\hat{e}}^{2}}_{ij}$, and ${{\hat{e}}^{2}}_{ij}$ represent the residuals of the panel regression model.

### 3.5 Panel long-run parameter estimations

The existence of cointegration among variables requires the need to examine the variables’ long-term relationship. We used two different estimators to find the long-run relationship and estimate the coefficients of explanatory variables. We used the AMG estimator for calculating long-run parameters, which was first introduced by Eberhardt and Bond [74]. Although the AMG technique is successful in the presence of cross-sectional dependence, its results for panel data with heterogeneity tend to be outstanding [75]. Furthermore, the issue of stationarity keeps it stable [70]. The AMG estimator is a long-run estimator built for a moderate number of cross-sections and periods that produce accurate results and assist in correcting works in the presence of panel heterogeneity and multifactor error terms [76]. AMG also has the advantage of using time-invariant fixed effects in the model. A standard dynamic effect parameter is also included. The process for this estimator is two-fold:

### AMG-Step 1

$$\Delta y_{it}=a_{i}+b_{i}\Delta x_{it}+c_{i}f_{t}+\sum_{t=2}^{T}\delta_{i}\Delta D_{t}+\varepsilon_{it}$$ (9)

### AMG-Step 2


$${\hat{b}}_{AMG}=N^{-1}\sum_{i=1}^{N}{\hat{b}}_{i}$$ (10)

Where Δ signifies the differenced operator; *y*_*it*_ and *x*_*it*_ describe observables; *b*_*i*_ denotes country-specific estimation coefficients; *f*_*t*_ denotes the unobserved common factor with heterogeneous factor; *δ*_*i*_ shows the standard dynamic procedure and time dummies’ coefficient; ${\hat{b}}_{AMG}$ represents "the mean group estimator" for AMG; and *ε*_*it*_ and *a*_*i*_ define the error term and intercept, respectively.

Moreover, Eberhardt and Bond [74] determined that both AMG and CCEMG performed well in Monte Carlo simulations when cross-sectional dependence (multi-factor error terms) and root mean square errors were seen in panel data with non-stationary variables (combined or not). As a result, when using the AMG estimator, no pre-testing of variables for stationarity or cointegration is needed [75]. AMG estimator has been used in some previous studies, such as Destek and Sarkodie [75] and Dong et al. [69]; however, no study has used the AMG estimator for checking the long-run correlation in the current context. The effects from AMG estimators are then tested for robustness using the CCEMG method, similar to the argument in the first part of the estimation. The CCEMG procedure, proposed by Pesaran [77] and simplified by Kapetanios et al. [74], is helpful in the case of cross-sectional dependence. This estimator performs well when the data has panel heterogeneity and multifactor error words. Consequently, group averages of common effects and variables are used in a linear combination [69]. The following regression can be used to measure the CCEMG estimator:

$$X_{it}=\partial_{1i}+\forall_{1}Z_{it}+\emptyset_{i}p_{t}+\alpha_{i}{\bar{X}}_{it}+\gamma_{i}{\bar{Z}}_{it}+\varepsilon_{it}$$ (11)

Where, *X*_*it*_ and *Z*_*it*_ are measurable variables, *p*_*t*_ is an unobservable common factor with heterogeneous coefficients, ∀_1_ is the country-specific estimates coefficient, ∂_1*i*_ and *ε*_*it*_ are the model’s intercept and error term, respectively.

### 3.6 Panel causality test

The study employed the Dumitrescu and Hurlin (D-H) panel causality test in our research. D-H test was introduced by Dumitrescu and Hurlin [78]. The equation for the D-H causality test as under:

$$y_{it}=a_{i}+\sum_{j-1}^{K}{\mu^{j}}_{i}(y_{i(t-j)}+\sum_{j-1}^{K}{\gamma^{j}}_{i}(x_{i(t-j)}+\varepsilon_{it}$$ (12)

Where *y* and *x* show observables; *μ*^*j*^_*i*_ indicate the autoregressive parameters and *γ*^*j*^_*i*_ show the regression coefficients. According to the null hypothesis, there is no causality in the panel. Alternative hypotheses show causality in the smallest cross-section element. We can test our hypothesis based on an average Wald statistic as presented in the following equation:

$$W_{N.T}^{HNC}=N^{-1}\sum_{i=1}^{N}W_{i.T}$$ (13)

## 4. Results and discussion

### 4.1 Descriptive statistics

**Fig 2:**
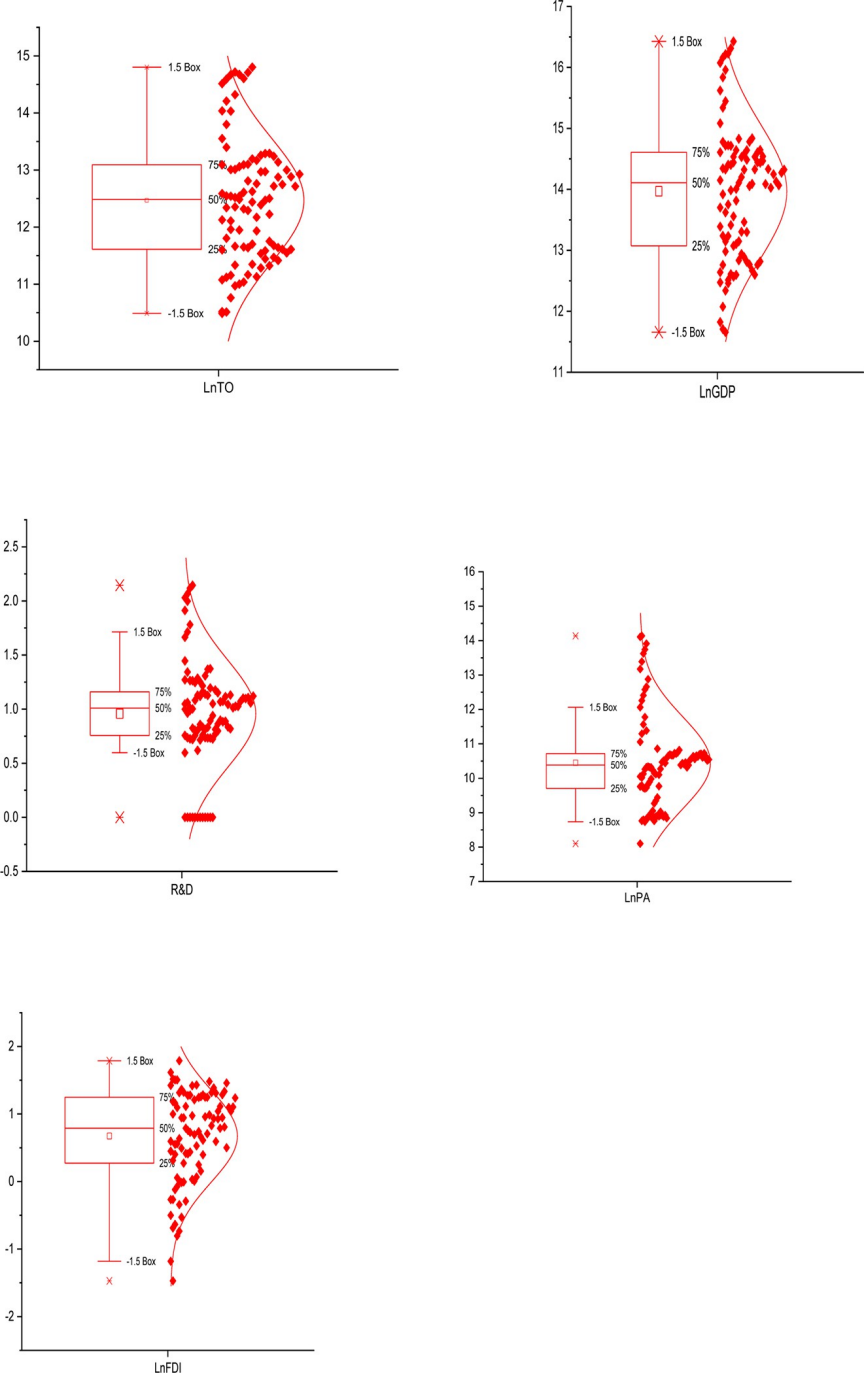
Box chart of the five variables. Note: The square signifies the mean values, the horizontal bar in the box indicates the median values, the dot represents the minimum/maximum values, and the top and bottom edges of the box represent the 75th percentile and 25th percentile, respectively.

Table 2 provides a glance of the nature of data. The average patent value throughout the panel is 113940.7, while the maximum value of a patent in a BRICS nation is 1381594, with a significantly high standard deviation, which explains the massive disparity of the values across the BRICS countries. Furthermore, FDI has a maximum value of 5.983% and a minimum value of 0.229%. The difference between the maximum and minimum values of the variables indicate significant disparities. The average per capita GDPPC is 2130475 US dollars, with a maximum GDPPC of 1.36E+07 US dollars and 115481.9 US dollars. Lastly, R&D spending ranges from a maximum of 2.145 to a low of 0.00006, with an average value of 0.958. Furthermore, Fig 2 shows the dispersed charting of BRICS nations’ R&D spending, FDI, GDPPC, trade openness, and patent applications.

**Table 2:** Descriptive statistics.

Variable		Mean	SD	Minimum	Maximum	Observation
*PAT*	overall	113940.7	263168.7	3295	1381594	N = 100
* *	between		198469.4	6946.789	468335.4	n = 5
* *	within		193417.9	-302488.7	1027199	T = 20
*FDI*	overall	2.369465	1.278969	0.2294564	5.983101	N = 100
* *	between		0.8081195	1.581701	3.253314	n = 5
* *	within		1.0525	0.5731635	6.770865	T = 20
*TO*	overall	483816.9	639907.7	35908.87	2684284	N = 100
* *	between		568065.4	85103.03	1481875	n = 5
* *	within		385456.8	-718497	1686226	T = 20
*GDPPC*	overall	2130475	2813806	115481.9	1.36E+07	N = 100
* *	between		2278176	285524.8	6105604	n = 5
* *	within		1929059	-2763798	9633023	T = 20
*R&D*	overall	0.9579905	0.4943999	0.00006	2.14512	N = 100
* *	between		0.3735512	0.5622042	1.481597	n = 5
* *	within		0.3627828	-0.5235468	1.621513	T = 20

Note: SD indicates standard deviation.

### 4.2 Outcomes of cross-sectional dependence

We have shown the outcomes of cross-sectional dependency among the underlying variables in Table 3. According to the results, the null hypothesis of cross-sectional independence is rejected at the 1% significance level, indicating a cross-sectional correlation between the variables.

**Table 3:** Estimation of cross-sectional dependence and slope homogeneity.

Variable	P CD	PS LM	B-P LM
*PAT*	9.073***	17.351***	87.597***
*FDI*	0.744	1.8717*	18.3705**
*TO*	12.937***	35.274***	167.751***
*GDPPC*	11.817***	29.292***	140.999***
*R&D*	3.928***	8.925***	49.914***
*Test for slope homogeneity (Pesaran*, *Yamagata*. *2008)*			
*Delta*	p-value	Adj. *Delta*	p-value
*3*.*618*	0.000	4.000	0.000

Note: P CD = Pesaran CD, PS LM = Pesaran scaled LM, B-G LM = Breusch-Pagan LM

** and *** indicate significance at the 5% and 1% level respectively, null hypothesis = no cross-sectional dependence

### 4.3 Outcomes of panel unit root test

Using the Pesaran (CIPS) unit root test, we have confirmed the underlying research variables’ stationarity level. Table 4 shows the results of the Pesaran (CIPS) unit root test. According to the obtained results, the null hypothesis is rejected level, with intercept, intercept, and trend. At first difference, stationarity was observed at a 1% significance level for intercept and intercept & trend. This shows the integration of underlying variables at the order I. Hence, the study variables are integrated at the order I (1), which justifies the investigation of long-run relationships.

**Table 4:** Pesaran CIPS Panel unit root test.

Variable	Level		First difference		Integration order
	Constant	Constant & trend	Constant	Constant & trend	
*PAT*	-1.126	-2.658	-4.106	-3.909	I[1]
*FDI*	-2.788	-2.792	-5.131	-5.389	I[1]
*TO*	-1.900	-3.319	-3.179	-3.319	I[1]
*GDPPC*	-1.545	-1.569	-2.512	-3.738	I[1]
*R&D*	-1.863	-1.478	-3.103	-3.257	I[1]

Note: critical values at 10%, 5% and 1% including constant; -2.21, -2.34, -2.6; and including constant & trend are; -2.74, -2.88, and -3.15 respectively.

### 4.4 Outcomes of panel cointegration test

We use Pedroni [79] and Kao [73] to determine the cointegration relation between variables, as shown in Table 5. According to the Pedroni test, the variables in this study seem to be relevant. This means that there is cointegration between variables. The results of the Kao test also show that there is a cointegration link between the primary variables of our model. Therefore, the Pedroni and Kao test results can guide estimating long-term relationships between variables. Therefore, use AMG and CCEMG for evaluation to determine long-term relationships.

**Table 5:** Panel co-integration results.

Ho: No co-integration Ha: All panels are co-integrated	Statistic	p-value(s)
Pedroni-cointegration		
Modified Phillips-Perron t-statistics	1.4532	0.0731
Phillips-Perron t-statistics	-2.4432	0.0073
Augmented Dickey-Fuller t-statistics	-1.4676	0.0711
Kao-cointegration		
Modified Dickey-Fuller (MDF) t-statistics	-6.4828	0.0000
Dickey-Fuller(DF) t-statistics	-2.7728	0.0028
Augmented Dickey-Fuller (ADF) t-statistics	-4.3619	0.0000
Unadjusted modified Dickey-Fuller (UMDF) t-statistics	-6.8042	0.0000
Unadjusted Dickey-Fuller(UDF) t-statistics	-2.8306	0.0023

Note: Ho is the null hypothesis, Ha is an alternative hypothesis and significance level at 5% and 1%.

### 4.5 Outcomes of panel AMG estimator

We have used an AMG estimator to investigate the impact of FDI, trade openness, GDP per capita, and R&D expenditure on technological innovation. Table 6 shows that LnFDI, LnTO, LnGDP, and LnR&D coefficients significantly impact technological innovation. The positive association between FDI and technological innovation shows that the FDI will increase by 1% in the long run, and the average technological innovation will increase by 0.21%. According to theory, competition and spillover effects from the foreign direct investment will affect how well innovations perform. First, FDI offers modern technology, equipment, and managerial expertise in addition to cash, which may have a ripple impact on other areas of the economy, such as demonstration, learning, and employee turnover. Therefore, FDI helps local businesses perform better in terms of innovation [28]. For instance, with the introduction of foreign-funded businesses, local businesses may mimic and raise their investment in R&D by learning the technology and management expertise of inflows of FDI technologies [80]. Our research results are similar to Hsu and Tiao [63] and Cheung and Lin [60]. Likewise, a positive link between trade openness and technological innovation indicates that a 1% increase in trade openness can drive technological innovation by 0.917. Trade openness is said to provide several economic advantages, such as greater technology transfer, talent transfer, increased labor and total factor productivity, and economic growth and development, providing such an opportunity is termed to improve technological innovations. An article appeared in “The Center for Economic Policy Research (CEPR)”, written by two professors of economics from Princeton and Harvard universities, (Redding & Melitz) [81] stated that market size that companies can reach grows as a result of international commerce. This increase in market size might increase the motivation to innovate to the degree that it includes fixed expenses that can be distributed over more production units. These results are similar to the mainstream literature on trade openness, such as Bhattacharya and Bloch [65] and Thanh et al. [82], but opposite to the results of Phuc et al. [83]. The coefficient GDP is 0.297, which means that a 1% GDP growth increases technological innovation by an average of 0.297% in the BRICS countries. Avila-Lopez et al. [24] also found similar results. Likewise, a 1% increase in R&D spending increases technological innovation by an average of 0.357%. Our results are consistent with the findings of Sierotowicz [84] and Meo and Usmani [85]. We can conclude that the estimation results show that an increase in FDI leads to a rise in technological innovation. Second, trade openness will also stimulate technological innovation through patent applications. Third, GDP has a significant positive impact on technological innovation. Finally, R&D spending plays a substantial role in expanding innovation in BRICS countries.

**Table 6:** Results of panel AMG and CCEMG estimators.

Variables	AMG(*LnPAT)*	CCEMG(*LnPAT*)
*FDI*	0.02101*** (0.0045)	0.0366*** (0.0171)
*LnTO*	0.9172*** (0.0531)	0.9588* (0.5587)
*LnR&D*	0.3575943* (0.2046)	0.02464 (0.0632)
*LnGDPPC*	0.2975*** (0.1315)	0.8107* (0.4253)
Observations	95	95
Groups	5	5
Wald χ-statistics(Prob > χ^2^)	326.31(0.000)	13.99(0.007)
Root mean squared error (RMSE)	0.1056	0.0509

Note: Standard errors in parentheses

*** p<0.01

** p<0.05

* p<0.1

### 4.6 Robustness analysis

We used the CCEMG estimator to test the robustness of our model. CCEMG results are consistent with AMG estimates. We found a significant positive impact of FDI, trade openness, GDP, and R&D spending on technological innovation in the BRICS panel. Thus, the study concludes that many factors driving innovation in the BRICS countries, including FDI, trade openness, and R&D spending, have a significant positive impact on technological innovation.

### 4.7 Results of Dumitrescu-Hurlin causality test

**Fig 3:**
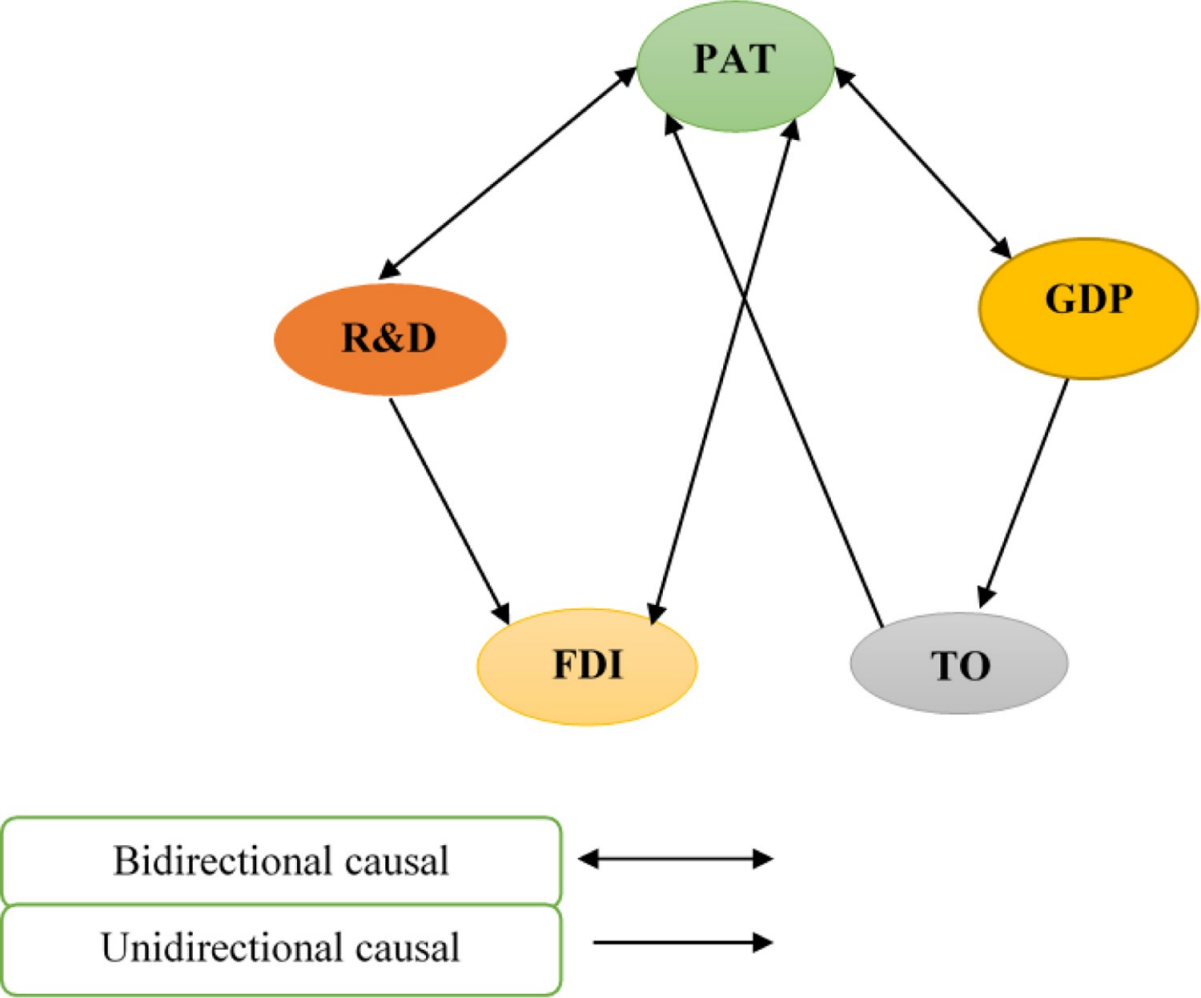
Flows of short-run causality relationship.

To test causality among the leading study variables, we used the Dumitrescu-Herlin causality test. Table 7 and Fig 3 show the causal relationship between variables for the BRICS countries. The results show a bi-directional causal relationship from GDP, R&D spending, and FDI to technological innovation. There is also a unidirectional causal relationship from GDP to trade openness and trade openness to technological innovation. Moreover, a one-way causal relationship is found between R&D spending and FDI.

**Table 7:** Results of Dumitrescu Hurlin panel causality tests.

Null Hypothesis:	W-Stat.	Zbar-Stat.	Prob.
* LnR&D ≠ LnPAT*	4.65515	1.66920	0.0870
* LnPAT* ≠ *LnR&D*	6.01020	3.04106	0.0073
* LnTO ≠ LnPAT*	8.21233	4.42159	1.E-05
* LnPAT ≠ LnTO*	2.60910	0.15907	0.8736
* LnGDPPC ≠ LnPAT*	7.39816	3.76296	0.0002
* LnPAT ≠ LnGDPPC*	7.76442	4.03919	5.E-05
* LnFDI ≠ LnPAT*	7.87668	4.12386	4.E-05
* LnPAT ≠ LnFDI*	9.13033	5.06937	4.E-07
* LnTO ≠ LNRD*	2.43585	0.02727	0.9782
* LnR&D ≠ LnTO*	2.24249	-0.11982	0.9046
* LnGDPPC ≠ LnR&D*	4.06189	1.24671	0.2125
* LnR&D ≠ LnGDPPC*	4.07179	1.25417	0.2098
* LnFDI ≠ LnR&D*	2.29610	-0.08507	0.9322
* LnR&D ≠ LnFDI*	4.75465	1.76919	0.0769
* LnGDPPC ≠ LnTO*	5.90919	2.63996	0.0083
* LnTO ≠ LnGDPPC*	2.97929	0.43020	0.6670
* LnFDI ≠ LnTO*	3.08735	0.51170	0.6089
* LnTO ≠ LnFDI*	3.05876	0.49014	0.6240
* LnFDI ≠ LnGDPPC*	3.43321	0.77255	0.4398
* LnGDPPC ≠ LnFDI*	4.51197	1.58616	0.1127

Note: The Dumitrescu Hurlin test is estimated with 2 lag and Zbar-statistics, LnX ≠ LnY suggests that Ln X does not homogeneously cause LnY

## 5. Conclusion and policy implications

### 5.1 Conclusion

Outbound foreign direct investment has received more attention from studies in international business and strategy domains in recent years. Although the causes and effects of outbound FDI have received a great deal of attention, less is known about inward FDI, its effects on the host nations, and particularly how it impacts the innovativeness of the host country. In line with this argument, we analyzed how an FDI inflow fostered technological innovations in the BRICS from 2000 to 2020. The study applied updated econometric approaches such as panel unit root, cointegration, and causality tests, which specialize attention to cross-sectional dependence, and panel heterogeneity. The empirical results from Pesaran’s cross-sectional dependence test stated that there is a strong cross-sectional dependence among countries due to the continuous improvement of economic interdependence. The results from long-run estimators (AMG and CCEMG) state that an increase in FDI leads to technological innovation. Second, trade openness will also stimulate technological innovation through patent applications. Third, GDP has a significant positive impact on technological innovation. Finally, R&D spending plays a significant role in expanding innovation in BRICS countries. Furthermore, the findings of this study also show that FDI, trade openness, GDPPC, and R&D expenditure significantly impact technological innovation in the BRICS countries. The causal results explored the two-way causality from GDPPC, R&D expenditure, and FDI to technological innovation. A one-way causal relationship exists between GDPPC and trade openness and technological innovation. Also, one-way causality is explored from R&D expenditure to FDI. This relationship shows that FDI, trade openness, GDPPC, and R&D expenditure stimulate technological innovation in the BRICS countries.

### 5.2. Policy implications

This research will help policymakers and governments in formulating policies. First, the FDI and trade openness contributes significantly to technological innovations, thus indicating that they are positive indicators of technological innovations in BRICS countries. We suggest that countries with insufficient technology should increase FDI to improve innovation based on the research results. Policymakers should formulate effective policies to attract more FDI, which catalysis innovation. The government also needs to increase trade volumes to promote regional innovation. Also, strict guidelines are required to increase the share of R&D expenditures to stimulate technological innovation in the BRICS countries.

### 5.3 Suggestions for future studies

This study is limited to BRICS countries, and the same study could be done in other groups of countries. Comparative analysis between the BRICS block and other blocks may be conducted. Future studies can consider green technological innovation along with the current variables.
